# Supplementary figures and images for: Rictor/TORC2 mediates gut-to-brain signaling in the regulation of phenotypic plasticity in C. elegans
Source: PLoS Genet. 2018 Feb 7;14(2):e1007213. doi: 10.1371/journal.pgen.1007213 (PMC5819832; doi:10.1371/journal.pgen.1007213)

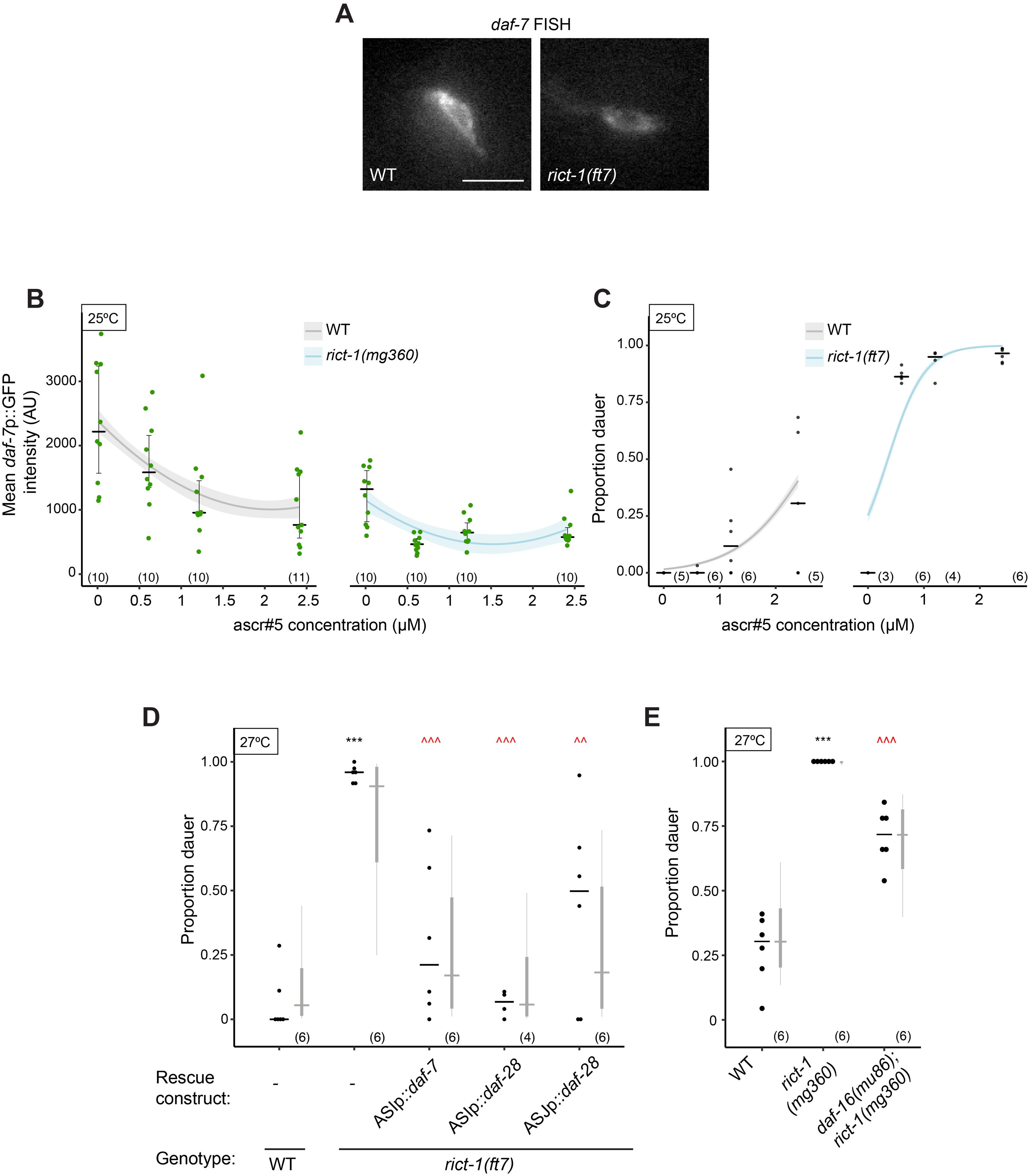

Supplement: S1 Fig — A) Representative images of daf-7 mRNA FISH in ASI neurons of WT and rict-1 mutant animals grown at 27°C. Scale bar: 5 μm. B) Quantification of daf-7p::gfp expression in ASI of wild-type (black) and rict-1(mg360) (blue) mutant animals at 25°C in the presence of the indicated concentrations of ascr#5. Each green dot is the mean fluorescence intensity in a single animal (2 neurons per animal); numbers in parentheses below indicate the number of animals examined in a single experiment with 2 biological replicates per genotype. Horizontal thick bar indicates median. Error bars are quartiles. Lines and shaded regions indicate mean and standard error predictions, respectively from a quadratic regression fit. For genotype, F = 39.9, Df = 1, P<0.001; for pheromone F = 15.845, Df = 2, P<0.001. C) Dauers formed by wild-type (black) and rict-1(ft7) (blue) animals at 25°C in the presence of the indicated concentrations of ascr#5. Each dot indicates the proportion of dauers formed in a single assay. Horizontal bar indicates median. Numbers in parentheses below indicate the number of independent experiments with at least 27 animals each. Lines indicate predictions from GLM fit, corresponding to an odds ratio of ~21.3 for rict-1 across this range of ascr#5 concentrations. For genotype, Wald X2 = 544, Df = 1, p < 0.0001; for pheromone, Wald X2 = 332, Df = 1, p < 0.0001 with GLM fit. D) Dauers formed by animals of the indicated genotypes at 27°C. Each black dot indicates the average number of dauers formed in a single assay. Horizontal black bar indicates median. Light gray thin and thick vertical bars at right indicate Bayesian 95% and 75% credible intervals, respectively. Numbers in parentheses below indicate the number of independent experiments with at least 36 and 9 animals each scored for non-transgenic and transgenic animals, respectively. Promoters driving expression in ASI and ASJ were srg-47p and trx-1p, respectively. One transgenic line was tested for each condition. ***— [file pgen.1007213.s001.tif]

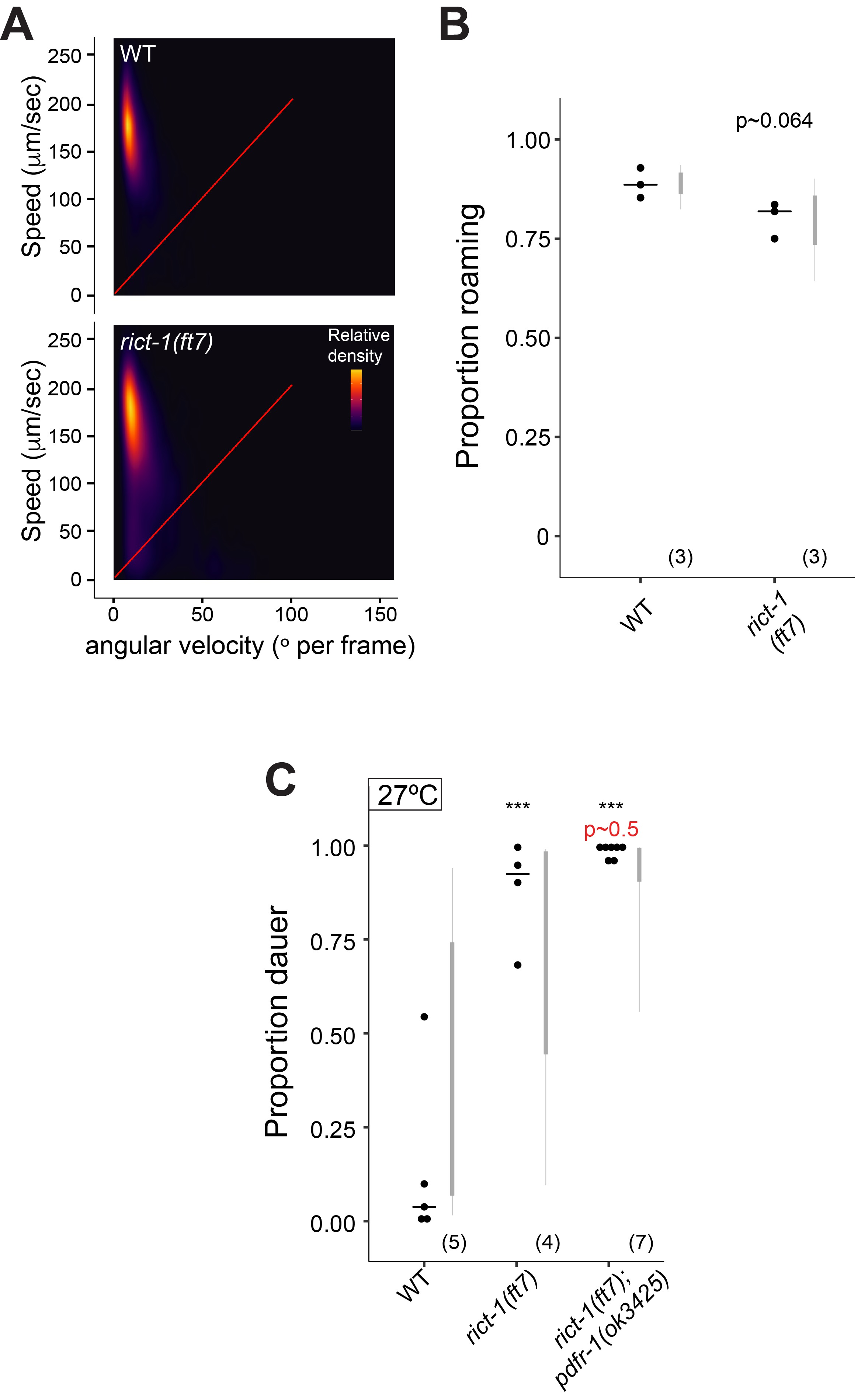

Supplement: S2 Fig — A) Live tracking of foraging behavior in starved worms. Shown is a representative assay. n > 20 animals per assay. Density plot shows mean speed and angular velocity from tracks binned over 10 sec intervals imaged at 3 frames/sec. Red line indicates delineation of roaming and dwelling behavior (slope = 2). B) Quantification of roaming and dwelling states. Dots show proportion of track time bins in which animals were roaming, with each dot reflecting one assay. Light gray thin and thick vertical bars at right indicate Bayesian 95% and 75% credible intervals, respectively. Numbers in parentheses below indicate the number of independent experiments with 20 animals each. P-value shown is with respect to wild-type (Welch’s t-test). C) pdfr-1 mutations do not suppress rict-1 dauer formation phenotypes. Each dot indicates the average number of dauers formed in a single assay. Horizontal bar indicates median. Light gray thin and thick vertical bars at right indicate Bayesian 95% and 75% credible intervals, respectively. Numbers in parentheses below indicate the number of independent assays with at least 27 animals each. P-value shown in comparison to rict-1 mutants. (TIF) [file pgen.1007213.s002.tif]

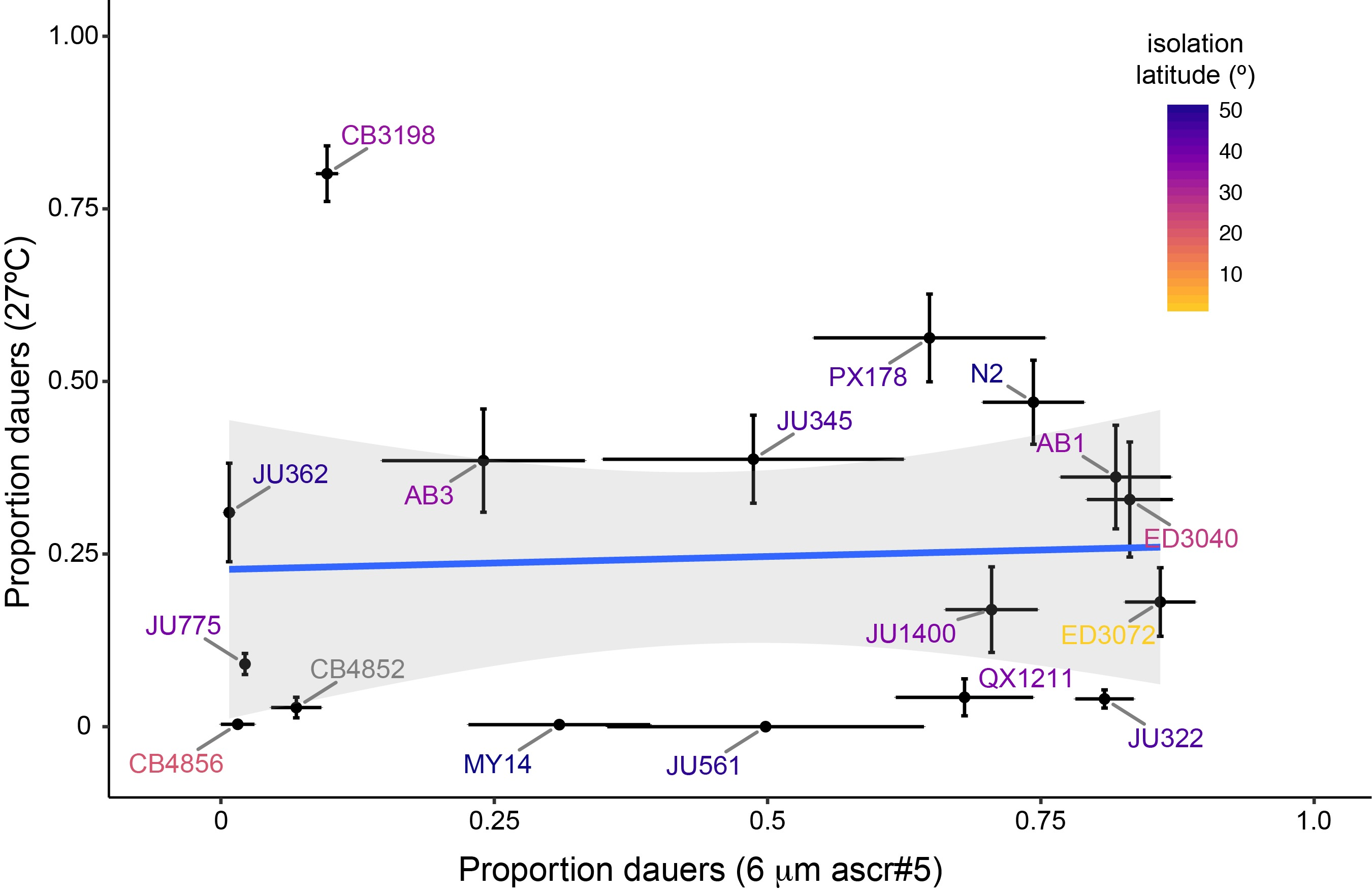

Supplement: S3 Fig — Shown are the proportion of dauers formed at 27°C and in the presence of 6 μm ascr#5 pheromone by C. elegans strains isolated from different latitudes. Assays were performed on live OP50. For ascr#5 data (X-axis), each data point is the average of at least 3 independent assays of at least 23 animals each. 27°C data are repeated from Fig 5A. Error bars are the SEM. Line and shaded region indicate linear regression fit using mean dauer formation values for each strain. (TIF) [file pgen.1007213.s003.tif]

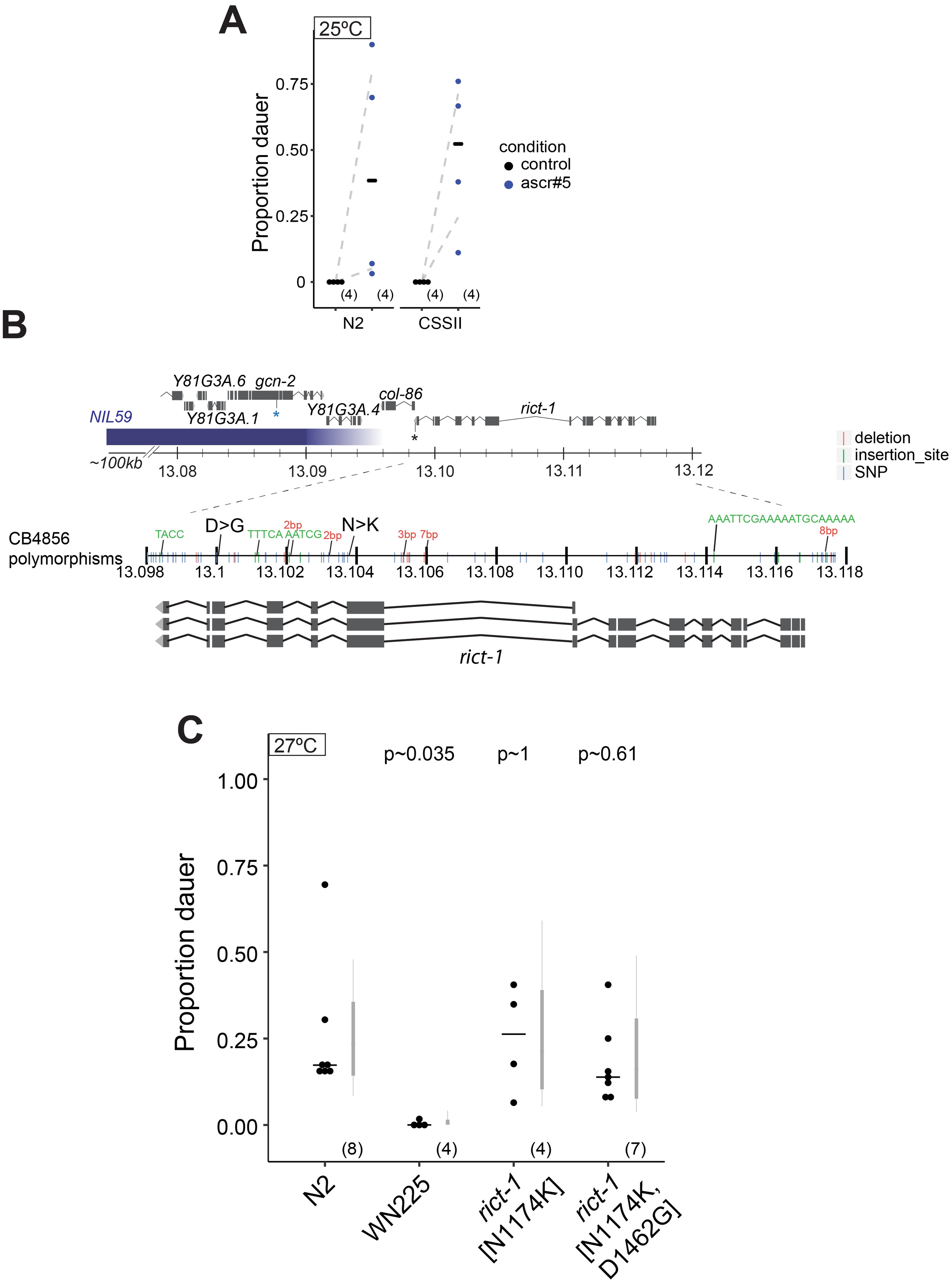

Supplement: S4 Fig — A) Pheromone-induced dauer formation is not reduced in the CSSII strain. Each dot indicates the average number of dauers formed in a single assay. Horizontal bar indicates median. Numbers in parentheses below indicate the number of independent experiments with at least 40 animals each. B) Estimated breakpoints of NIL59, the smallest interval containing QTL1. Top panel shows gene models near the right breakpoint of NIL59, indicated with blue bar. The right breakpoint lies between a polymorphism in gcn-2 (indicated with blue asterisk), and a polymorphism in the 3’UTR of rict-1 (indicated with black asterisk), and thus does not include the rict-1 coding region. Bottom panel shows all CB4856 polymorphisms in rict-1, with missense mutations indicated in black text. C) Dauers formed at 27°C by N2, the WN225 NIL, and strains in which the indicated polymorphisms in CB4856 rict-1 coding sequences have been introduced into N2 rict-1 sequences via gene editing. Each dot indicates the average number of dauers formed in a single assay. Horizontal bar indicates median. Light-grey thick and thin vertical bars indicate Bayesian 95% and 75% credible intervals, respectively. Numbers in parentheses below indicate the number of independent experiments with at least 40 animals each. P-values are relative to N2 (ANOVA with Dunnett-type multivariate-t post-hoc adjustment). (TIF) [file pgen.1007213.s004.tif]
