# Supplementary material for: Rictor/TORC2 mediates gut-to-brain signaling in the regulation of phenotypic plasticity in C. elegans
Source: PLoS Genet. 2018 Feb 7;14(2):e1007213. doi: 10.1371/journal.pgen.1007213 (PMC5819832; doi:10.1371/journal.pgen.1007213)
Supplement: S1 Table — (DOCX) [file pgen.1007213.s005.docx]

**Table S1.** List of strains used in this work.

| Strain | Genotype | Source and/or  parent strains^a^ | Relevant Figures |
| --- | --- | --- | --- |
| WT | N2 (Bristol) | CGC | all |
| KQ6 | *rict-1(mg360)* II | CGC | 1B-C, 2A-B, 2D, S1B, S1E |
| KQ1366 | *rict-1(ft7)* II | CGC | 1B-C, 2A-D, 3E-F, 4B-F, S1A, S1C-D, S2 |
| KQ1564 | *sgk-1(ok538)* X | CGC | 1B-C |
| BQ1 | *akt-1(mg306)* V | CGC | 1B-C |
| VC204 | *akt-2(ok393)* X | CGC | 1B |
| VC127 | *pkc-2(ok328)* X | CGC | 1B |
| FX01996 | *pdf-1(tm1996)* III | NBRP | 4E |
| VC2609 | *pdfr-1(ok3425)* III | CGC | 4E |
| PY9341 | *pdf-2(tm4394)* X | LSC59 (Meelkop et al., 2012) - lstEx24 crossed out. | 4F |
| CB4856 | WT - CB4856 (Hawaiian) | J.K. | 5A-B, 6, S3 |
| JU561 | WT - JU561 | CGC | 5A-B, S3 |
| MY14 | WT - MY14 | CGC | 5A-B, S3 |
| DL238 | WT- DL238 | CGC | 5A, S3 |
| CB4852 | WT - CB4852 | CGC | 5A, S3 |
| QX1211 | WT - QX1211 | CGC | 5A, S3 |
| JU322 | WT - JU322 | CGC | 5A-B, S3 |
| JU775 | WT - JU775 | CGC | 5A, S3 |
| JU1400 | WT - JU1400 | CGC | 5A, S3 |
| ED3072 | WT - ED3072 | CGC | 5A, S3 |
| AB1 | WT - AB1 | CGC | 5A, S3 |
| AB3 | WT - AB3 | CGC | 5A, S3 |
| JU362 | WT - JU362 | CGC | 5A-B, S3 |
| ED3040 | WT - ED3040 | CGC | 5A-B, S3 |
| JU345 | WT - JU345 | CGC | 5A-B, S3 |
| PX178 | WT - PX178 | CGC | 5A-B, S3 |
| CB3198 | WT - CB3198 | CGC | 5A-B, S3 |
| PY9323 | *rict-1(ft7)* II; *pdfr-1(ok3425)* III | KQ1366, VC2609 | 4E, S2C |
| PY9324 | *daf-16(mu86)* I; *rict-1(ft7)* II | CF1139 (Lin et al.,2001) - muIs61 outcrossed, KQ1366 | S1E |
| PY9349 | *rict-1(ft7)* II; *pdf-2(tm4393)* X | KQ1366, LSC59 - lstEx24 outcrossed | 4F |
| PY9350 | *rict-1(ft7)* II; lstEx24[*pdf-2*p::*pdf-2*] | KQ1366, LSC59 - *pdf-2(tm4393)* outcrossed | 4F |
| PY9351 | lstEx24[*pdf-2*p::*pdf-2*] | LSC59 - *pdf-2(tm4393)* outcrossed | 4F |
| PY9325 | *rict-1(mg360)* II; Ex[*ges-1p::rict-1::SL2::mCherry unc-122p::GFP*] line 1 | injected into KQ6 | 1C, 2B |
| PY9326 | *rict-1(mg360)* II; Ex[*gpa-4p::rict-1::SL2::mCherry unc-122p::GFP*] line 1 | injected into KQ6 | 1C |
| PY9327 | *rict-1(mg360)* II; Ex[*gpa-4p::rict-1::SL2::mCherry unc-122p::GFP*] line 2 | injected into KQ6 | 1C |
| PY9313 | *rict-1(ft7)* II; Ex[*ges-1p::rict-1::SL2::mCherry unc-122p::GFP*] line 2 | injected into KQ1366 | 1C, 4B |
| PY9314 | *rict-1(ft7)* II; Ex[*elt-2p::rict-1::SL2::mCherry unc-122p::GFP*] line 1 | injected into KQ1366 | 1C |
| PY9315 | *rict-1(ft7)* II; Ex[*gpa-4p::rict-1::SL2::mCherry unc-122p::GFP*] line 3 | injected into KQ1366 | 1C |
| PY9316 | *rict-1(ft7)* II; Ex[*ifb-2p::rict-1::SL2::mCherry unc-122p::GFP*] line 1 | injected into KQ1366 | 1C |
| PY9319 | *rict-1(ft7)* II; Ex[*gpa-4p::rict-1::SL2::mCherry unc-122p::GFP*] line 4 | injected into KQ1366 | 1C |
| PY9320 | *rict-1(ft7)* II; Ex[*ifb-2p::rict-1::SL2::mCherry unc-122p::GFP*] line 2 | injected into KQ1366 | 1C |
| PY9322 | *rict-1(ft7)* II; Ex[*elt-2p::rict-1::SL2::mCherry unc-122p::GFP*] line 2 | injected into KQ1366 | 1C |
| PY9328 | *rict-1(ft7)* II; Ex[*rict-1p::rict-1::SL2::mCherry unc-122p::GFP*] line 1 | injected into KQ61366 | 4B |
| PY9329 | *sgk-1(ok538)* X; Ex[*ges-1p::sgk-1b::SL2::mCherry unc-122p::GFP*] line 1 | injected into KQ1564 | 1C |
| PY9330 | *akt-1(mg306)*; Ex[*ges-1p::akt-1b::SL2::mCherry unc-122p::GFP*] line 1 | injected into BQ1 | 1C |
| FK181 | *ksIs2[daf-7p::gfp rol-6*(*su1006*)*]* | (Murakami et al., 2001) | 2A, 3A, S1B |
| PY9331 | *rict-1(mg360)* II; *ksIs2[daf-7p::gfp rol-6*(*su1006*)] | FK181, KQ6 | 2A, S1B |
| PY9332 | *rict-1(ft7)* II; *ksIs2[daf-7p::gfp rol-6*(*su1006*)] | FK181, KQ1366 | 2A |
| PY9333 | *rict-1(ft7)* II; *ksIs2*[*daf-7p::gfp rol-6*(*su1006*)]; Ex[*ges-1p::rict-1::SL2::mCherry* *unc-122p::GFP*] line 3 | injected into PY9332 | 2A |
| PY9342 | *rict-1(ft7)* II; *ksIs2*[*daf-7p::gfp rol-6*(*su1006*)]; Ex[*ifb-2p::rict-1::SL2::mCherry* *unc-122p::mCherry*] line 3 | injected into PY9332 | 2A |
| PY9343 | *rict-1(ft7)* II; *ksIs2*[*daf-7p::gfp rol-6*(*su1006*)]; Ex[*gpa-4p::rict-1::SL2::mCherry* *unc-122p::mCherry*] line 5 | injected into PY9332 | 2A |
| PY9344 | *rict-1(ft7)* II; *ksIs2*[*daf-7p::gfp rol-6*(*su1006*)]; Ex[*gpa-4p::rict-1::SL2::mCherry* *unc-122p::mCherry*] line 6 | injected into PY9332 | 2A |
| GR1455 | *mgIs40*[*daf-28*p::NLS::*gfp* *lin-15*+] | CGC | 2C, 3B, 3E-F |
| PY9340 | *rict-1(ft7)* II; *mgIs40*[*daf-28*p::NLS::*gfp* *lin-15*+] | KQ1366, GR1455 | 2C, 3E-F |
| PY9345 | *rict-1(ft7)* II; *mgIs40*[*daf-28*p::NLS::*gfp* *lin-15*+]; Ex[*ifb-2*p*::rict-1::SL2::mCherry* *unc-122p::mCherry*] line 4 | injected into PY9340 | 2C |
| PY9346 | *rict-1(ft7)* II; *mgIs40*[*daf-28*p::NLS::*gfp* *lin-15*+]; Ex[*ifb-2*p*::rict-1::SL2::mCherry* *unc-122p::mCherry*] line 5 | injected into PY9340 | 2C |
| PY9347 | *rict-1(ft7)* II; *mgIs40*[*daf-28*p::NLS::*gfp* *lin-15*+]; Ex[*gpa-4*p*::rict-1::SL2::mCherry* *unc-122p::mCherry*] line 7 | injected into PY9340 | 2C |
| PY9348 | *rict-1(ft7)* II; *mgIs40*[*daf-28*p::NLS::*gfp* *lin-15*+]; Ex[*gpa-4*p*::rict-1::SL2::mCherry* *unc-122p::mCherry*] line 8 | injected into PY9340 | 2C |
| PY9334 | *rict-1(ft7)* II; Ex[*srg-47*p::*daf-7* *unc-122*p::*gfp*] line 1 | PY10737 (Neal et al., 2015), KQ1366 | S1D |
| PY9335 | *rict-1(ft7)* II; Ex[*srg-47*p::*daf-28*(gDNA) *unc-122*p::*gfp*] line 1 | PY10728 (Neal et al., 2015), KQ1366 | S1D |
| PY9336 | *rict-1(ft7)* II; Ex[*trx-1*p::*daf-28*(gDNA) *unc-122*p::*gfp*] line 1 | PY10730 (Neal et al., 2015), KQ1366 | S1D |
|  |  |  |  |
| WE5236 | *“CSSI” pgIR1 (I, CB4856 > N2)* | (Glauser et al., 2011) | 6A |
| GN67 | *“CSSII” pgIR2 (II, CB4856 > N2)* | (Glauser et al., 2011) | 6A |
| GN68 | *“CSSIII” pgIR3 (III, CB4856 > N2)* | (Glauser et al., 2011) | 6A |
| GN69 | *“CSSIV” pgIR4 (IV, CB4856 > N2)* | (Glauser et al., 2011) | 6A |
| GN70 | *“CSSV” pgIR5 (V, CB4856 > N2)* | (Glauser et al., 2011) | 6A |
| GN231 | *“CSSX” pgIR6 (X, CB4856 > N2)* | (Glauser et al., 2011) | 6A |
| WN219 | *ewIR19* *(II, CB4856 > N2)* | (Doroszuk et al., 2009) | 6C |
| WN220 | *ewIR20* *(II, CB4856 > N2)* | (Doroszuk et al., 2009) | 6C |
| WN221 | *ewIR21* *(II, CB4856 > N2)* | (Doroszuk et al., 2009) | 6C |
| WN222 | *ewIR22* *(II, CB4856 > N2)* | (Doroszuk et al., 2009) | 6C |
| WN223 | *ewIR23* *(II, CB4856 > N2)* | (Doroszuk et al., 2009) | 6C |
| WN224 | *ewIR24* *(II, CB4856 > N2)* | (Doroszuk et al., 2009) | 6C |
| WN225 | *ewIR25* *(II, CB4856 > N2)* | (Doroszuk et al., 2009) | 6C, 6F, S4C |
| WN226 | *ewIR26* *(II, CB4856 > N2)* | (Doroszuk et al., 2009) | 6C |
| WN227 | *ewIR27* *(II, CB4856 > N2)* | (Doroszuk et al., 2009) | 6C, 6F |
| PY9301 | *“NIL10” pyIR10 (II, CB4865 > N2)* | WN225, N2 | 6E |
| PY9304 | *“NIL59” pyIR59 (II, CB4865 > N2)* | WN225, N2 | 6E |
| PY9305 | *“NIL76” pyIR76 (II, CB4865 > N2)* | WN225, N2 | 6E |
| PY9306 | *“NIL78” pyIR78 (II, CB4865 > N2)* | WN225, N2 | 6E |
| PY9337 | *rict-1[N1174K]* II | N2 | S4C |
| PY9338 | *rict-1[N1174K,D1462G]* II | N2 | S4C |
| ^a^CGC – *Caenorhabditis* Genetics Center; NBRP – National BioResource Project | | | |
|  | | | |
