# Supplementary material for: Rictor/TORC2 mediates gut-to-brain signaling in the regulation of phenotypic plasticity in C. elegans
Source: PLoS Genet. 2018 Feb 7;14(2):e1007213. doi: 10.1371/journal.pgen.1007213 (PMC5819832; doi:10.1371/journal.pgen.1007213)
Supplement: S2 Table — (DOCX) [file pgen.1007213.s006.docx]

**Table S2.** Type-I and type-II error rates indicate Bayesian GLMM outperforms frequentist methods for variable dauer data.

| Type-I error estimates (low variance) | | | |  |  |  |  |  |  |  |  |  |
| --- | --- | --- | --- | --- | --- | --- | --- | --- | --- | --- | --- | --- |
| Model | β (C) | sP | sD | sG | t (G1) | t (G2) | ANOVA (G1) | ANOVA (G2) | GLMM (G1) | GLMM (G2) | STAN (G1) | STAN (G2) |
|  |  |  |  |  |  |  |  |  |  |  |  |  |
| C = G1 = G2 | 0 | 0.3 | 0 | 0 | 0.04 | 0.049 | 0.023 | 0.018 | 0.043 | 0.036 | NA | NA |
| C = G1 = G2 | -2.2 | 0.3 | 0 | 0 | 0.032 | 0.045 | 0.023 | 0.024 | 0.039 | 0.045 | NA | NA |
| C = G1 = G2 | 0 | 0.3 | 0.2 | 0.2 | 0.067 | 0.07 | 0.046 | 0.049 | 0.071 | 0.069 | NA | NA |
| C = G1 = G2 | -2.2 | 0.3 | 0.2 | 0.2 | 0.046 | 0.051 | 0.038 | 0.038 | 0.067 | 0.067 | NA | NA |
| C = G1 = G2 (u) | 0 | 0.3 | 0.2 | 0.2 | 0.065 | 0.054 | 0.045 | 0.029 | 0.05 | 0.041 | NA | NA |
| C = G1 = G2 (u) | -2.2 | 0.3 | 0.2 | 0.2 | 0.059 | 0.047 | 0.042 | 0.039 | 0.054 | 0.049 | NA | NA |
| C = G1 = G2 (b) | 0 | 0.3 | 0.2 | 0.2 | 0.049 | 0.056 | 0.134 | 0.164 | 0.071 | 0.078 | 0.033 | 0.039 |
| C = G1 = G2 (b) | -2.2 | 0.3 | 0.2 | 0.2 | 0.041 | 0.049 | 0.121 | 0.085 | 0.078 | 0.056 | 0.041 | 0.028 |
|  |  |  |  |  |  |  |  |  |  |  |  |  |
| Type-I and type-II error estimates (low variance) | | | | |  |  |  |  |  |  |  |  |
| Model | β (C) | sP | sD | sG | t (G1) | t (G2) | ANOVA (G1) | ANOVA (G2) | GLMM (G1) | GLMM (G2) | STAN (G1) | STAN (G2) |
|  |  |  |  |  |  |  |  |  |  |  |  |  |
| C = G1 < G2 | 0 | 0.3 | 0 | 0 | 0.04 | 0.702 | 0.055 | 0.66 | 0.096 | 0.763 | NA | NA |
| C = G1 < G2 | -2.2 | 0.3 | 0 | 0 | 0.038 | 0.583 | 0.021 | 0.648 | 0.1 | 0.693 | NA | NA |
| C = G1 < G2 | 0 | 0.3 | 0.2 | 0.2 | 0.062 | 0.896 | 0.08 | 0.898 | 0.122 | 0.925 | 0.018 | 0.786 |
| C = G1 < G2 | -2.2 | 0.3 | 0.2 | 0.2 | 0.047 | 0.803 | 0.027 | 0.838 | 0.088 | 0.868 | NA | NA |
| C = G1 < G2 (b) | 0 | 0.3 | 0.2 | 0.2 | 0.056 | 0.868 | 0.185 | 0.773 | 0.148 | 0.807 | 0.045 | 0.772 |
| C = G1 < G2 (b) | -2.2 | 0.3 | 0.2 | 0.2 | 0.042 | 0.737 | 0.087 | 0.758 | 0.133 | 0.733 | 0.042 | 0.694 |
|  |  |  |  |  |  |  |  |  |  |  |  |  |
| Type-I and type-II error estimates (high day and genotype-specific variance) | | | | | | |  |  |  |  |  |  |
| Model | β (C) | sP | sD | sG | t (G1) | t (G2) | ANOVA (G1) | ANOVA (G2) | GLMM (G1) | GLMM (G2) | STAN (G1) | STAN (G2) |
|  |  |  |  |  |  |  |  |  |  |  |  |  |
| C = G1 = G2 | 0 | 0.1 | 0.5 | 0.5 | 0.097 | 0.097 | 0.068 | 0.067 | 0.091 | 0.106 | 0.043 | 0.043 |
| C = G1 = G2 | -2.2 | 0.1 | 0.5 | 0.5 | 0.078 | 0.084 | 0.054 | 0.068 | 0.089 | 0.091 | 0.035 | 0.04 |
| C = G1 < G2 | 0 | 0.1 | 0.5 | 0.5 | 0.082 | 0.592 | 0.109 | 0.549 | 0.163 | 0.597 | 0.038 | 0.416 |
| C = G1 < G2 | -2.2 | 0.1 | 0.5 | 0.5 | 0.06 | 0.507 | 0.028 | 0.55 | 0.134 | 0.59 | 0.031 | 0.392 |
| C = G1 = G2 (b) | 0 | 0.1 | 0.5 | 0.5 | 0.157 | 0.153 | 0.138 | 0.138 | 0.098 | 0.102 | 0.058 | 0.062 |
| C = G1 = G2 (b) | -2.2 | 0.1 | 0.5 | 0.5 | 0.116 | 0.104 | 0.107 | 0.119 | 0.084 | 0.085 | 0.052 | 0.057 |
| C = G1 < G2 (b) | 0 | 0.1 | 0.5 | 0.5 | 0.136 | 0.713 | 0.166 | 0.742 | 0.15 | 0.654 | 0.061 | 0.596 |
| C = G1 < G2 (b) | -2.2 | 0.1 | 0.5 | 0.5 | 0.116 | 0.643 | 0.092 | 0.738 | 0.151 | 0.643 | 0.067 | 0.58 |

Clustered binary data were simulated as described in Statistical Analyses in Methods*.* For frequentist methods, type I error rates were summarized as the proportion of datasets with *P*<0.05 compared to the control group when group means were equal, and power as the proportion of datasets with *P*<0.05 when group means were different. Similarly, for Bayesian GLMM, the proportion of datasets in which the 95% credible interval for the group mean parameter estimate relative to the control does not include 0 are shown.

Red shading: Type I error rates > 0.06.

Blue shading: Power levels <60%.

*u*: Unbalanced models

*b*: Biased models.
